# Case of Strangulated Oblique Inguinal Hernia, in Which Several of the Diagnostic Signs Were Wanting; with Clinical Remarks

**Published:** 1827-01

**Authors:** 

**Affiliations:** St. Thomas's Hospital.


### STRANGULATED HERNIA.

*Case of Strangulated Oblique Inguinal Hernia, in which several of the Diagnostic Signs were wanting; with Clinical Remarks.* Treated by Mr. Tyrrell, at St. Thomas's Hospital.

December 5th, 1826.—William Poole, ætatis twenty, of athletic appearance, by trade a chimney-sweeper, accustomed to carry heavy weights, was admitted about six p.m. with an inflamed tumor in the right groin and scrotum. He stated that he had been the subject of hernia eight years, but had never worn a truss; that he had occasionally suffered attacks of pain in the tumor, attended with confinement of the bowels, which symptoms had always been speedily relieved by taking a little brandy and some aperient pills. Since Friday last the bowels have not been evacuated, and on Saturday (2d) he was seized with pain in the tumor. The following morning, irritability of the stomach ensued, which continued up to the time of his admission, notwithstanding the employment of the remedies which had on former occasions afforded him relief.

To-day, when admitted, the tumor was tense, painful, and tender, but not œdematous; the scrotal surface red and shining, and at the lower part slight fluctuation was perceptible. The swelling did not increase immediately after leaving the external abdominal ring, but a space of about one inch intervened between the ring and the tumor. This space, and also the inguinal canal as high as the internal ring, were hard and incompressible, and did not dilate on coughing. The patient vomited very frequently, with scarcely any effort whatever, and complained of tightness across the abdomen; which was, however, neither very tense nor very tender. The pulse was about 100, small, but without the threadiness usually attendant on peritoneal inflammation. By direction of the dresser, he was placed in a warm bath, and, becoming faint, the taxis was attempted, but without avail. Mr. Tyrrell was now sent for, who, after an attentive consideration of the symptoms and of the appearance of the tumor, thought that it more resembled inflammation of the testicle than strangulated hernia; and that the irritability of the stomach was the consequence of its sympathy with the inflammation of that organ. This opinion was strengthened by the concurrence of Mr. Calloway, who was invited to see the case. As apparent fluctuation existed at the lower part of the tumor, a puncture was made through the integument in that situation, which was followed only by a discharge of blood. As the symptoms were not urgent, it was then resolved to wait a few hours, and to endeavour to procure an evacuation from the bowels, which, if freely obtained, would disprove the existence of hernia.

A Castor-oil Enema was administered between seven and eight p.m. and repeated after two hours.—Applic^r Hirudines xx. abdomini et postea Fotus Papav. et Catap. Lini.

Twelve P.M.—Mr. Tyrrell, accompanied by Mr. Calloway, again visited the patient, and found him labouring under the same symptoms, without any aggravation. No evacuation had taken place; the countenance was not anxious, and there was no hiccough, but the vomiting continued: the ejected matter was offensive, but had no fœcal odour. An operation was now resolved upon, in order to determine the precise nature of the tumor, and, if hernia actually existed, to relieve the bowel from strangulation.

Dec. 6th, one A.M.—Mr. Tyrrell made an incision through the integuments and superficial fascia, beginning just above the external ring, and extending to the bottom of the scrotum. The cremaster muscle, much thickened, was now exposed, and, on endeavouring to raise it up, it was found to be firmly adherent to the part beneath: an opening was however made, and the muscle slit up on a director, when a hernial sac was exposed. On opening this sac, three or four drachms of serum escaped; proving that the puncture made last evening had merely penetrated the integuments. The other contents of the sac consisted of dark-coloured gangrenous omentum, and about four inches of the small intestines, of a dark chocolate colour, but not in a state of gangrene. On examination, a stricture was found at the external ring, which was divided; and, on passing the finger into the inguinal canal, another band of stricture was discovered, after the division of which the finger was again stopped at the internal ring: this was also freely divided. The omentum and the intestine were then ascertained to be adherent to the sac in the whole inguinal canal. After carefully separating the adhesions, the intestine was examined, when a gangrenous spot was perceived on the upper and lower portions at the point of stricture; not, however, so soft as to yield to moderate pressure. Conjecturing that the superior and internal portion communicated with the stomach, an opening was made into it, when some fluid buff-coloured matter escaped. The omentum was then removed by the knife: no bleeding ensued, the vessels being filled with coagulated blood. The intestine was left in the wound, over which a poultice was applied. As the irritability of the stomach continued, the following mixture was ordered:

℞. Magnes. Sulph. ʒ iij.; Aquæ Menth. Pip. ℥ jss.; Tr. Opii gtt. v. M. tertia quâque horâ sumend.

Six A.M.—The pulse has risen, and is harder; the vomiting continues; the discharge from the wound is free.

℞. Cal. gr. iij.; P. Opii gr. j. M. statim sumend.—V.S. ad ℥ xij.

After the abstraction of this quantity of blood, he became faint, and no more could be obtained. The blood exhibited the buffy coat.

Noon.—The rectum not having been relieved by the former injection, the following was ordered to be administered immediately:—

Enema Coloc. C.—Adde Mist. Tr. Opii gtt. v. (ut sumantur gtt. x.)

About twenty minutes after administering the enema, an evacuation took place, and after a few hours a second.

Vespere.—The pulse is rather sharp: the vomiting continues. Omit the former medicines, and take the following:

℞. Mist. Efferv.; Tr. Opii gtt. xv. M. statim sumend. et post horas duas repetend.—℞. Cal., P. Opii, āā gr. j. M. hàc nocte sumend.—V.S. ad ℥ viij. statim.

The blood drawn this evening was not so much buffed as the former. The patient has been dosing throughout the day.

Dec. 7th, mane.—He continues in much the same state as yesterday: the discharge from the groin is copious, rendering necessary the frequent renewal of the poultice. The vomiting does not recur so often, but this symptom appears to subside more from weakness than from any other cause.

Beef-tea ℔j. pro enemate.—℞. Cal., P. Opii āā gr. ss. M. secunda quâque horâ.—Rep$^{r}$ Mist. Efferv. p. r. n.

Vespere.—Pulse 104, small and sharp; abdomen neither tense nor tender.

V.S. e brachio ad ℥ iv.

During the abstraction of the blood, the pulse lost its sharpness. As the vomiting continues, substitute weak brandy and water for the Mist. Efferv.

8th.—Pulse 108, small and feeble. The vomiting continues, but is rather less frequent; the countenance is pallid, and the eyes rather sunk. He makes scarcely any complaint, and lies in a half-comatose state, from which he is easily roused, and answers questions with tolerable readiness. He continued gradually to sink, with hardly any alteration of the symptoms. Arrow-root, and eggs, and brandy, were administered at short intervals, which remained on the stomach about twenty minutes. As he regularly and progressively became more feeble, without an effort to rally, it was thought that probably the strangulated intestine was a portion of the jejunum, and that consequently no sufficient length of surface remained from which absorption of nutriment could take place adequate to the support of the system. Under this impression, one pound of beef-tea was injected into the rectum this evening.

℞. Pil. Cal. cum Opio hàc nocte sumend.

Dec. 9th.—Has continued to sink gradually, notwithstanding the administration of stimulants and nutritive enemata. Vomiting occurs at intervals of about two hours; pulse 110, small and feeble. He is perfectly sensible.

℞. Jusculi Bovini ℔ss.; Vini rubri ℥ ij.; Tr. Opii m. xv. M. fiat enema statim injiciendum et quartâ quâque horâ repetend.

At half-past ten P.M. he expired.

Thirteen hours after death, the body was examined by Mr. Tyrrell and Mr. Calloway, in the presence of a number of students.

The protruded intestine, of a dark leaden colour, adhered firmly to the lower three-fourths of the internal ring: it was perfectly free from stricture, and the finger could be readily passed from the opening of the intestine in the sac, into either the upper or lower portions of the canal in the abdomen. There was considerable thickening of the scrotum over the right testicle, which was slightly enlarged and indurated. On opening the abdomen, not a tea-spoonful of serum could be perceived in its cavity, and the peritoneum lining the parietes was quite pale and transparent. The ileum, at about the distance of a foot from its termination in the cœcum, was protruded through the internal ring, having on its inner side the epigastric artery. The peritoneal coat of this bowel exhibited an inflammatory blush, and, for about an inch and a half above the internal ring, the upper and lower portions were united at an acute angle by a thin layer of adhesive matter, which readily gave way on the application of very slight force. On opening the intestine, its mucous coat exhibited marks of acute inflammation, which extended into the colon and to a considerable distance up the ileum, the upper portion of the ileum and the jejunum being uninflamed. The contents of the small intestines were buff-coloured, of a pappy consistence, similar to the discharge from the groin. The colon was contracted, and contained a very small quantity of black softened fæces. The gall-bladder was full of greenish bile. The other viscera presented no peculiarity.

In a clinical lecture, delivered December 11th, Mr. Tyrrell made some observations, of which the following is the substance.

From such a case as the preceding, much more valuable information is to be derived, than from a number of cases in which the parts present the usual appearances, and in which the operation has been followed by the ordinary symptoms, and terminated favourably. At first I was induced to believe that the patient was not suffering from strangulated hernia. He had a sense of tightness across the abdomen, with vomiting, and the pulse was more frequent than natural, but it had neither the sharpness nor hardness indicating peritoneal inflammation; neither was there much tension nor tenderness of the abdomen, nor any anxiety of countenance. The tumor in the groin had not the usual appearances of hernia, but all those of common inflammation of the testicle. In hernia, the intestine usually forms a swelling immediately after passing the external ring, in consequence of the laxity of the parts permitting distention; but in this case there was a tumor at the lower part of the scrotum, and between this and the external ring intervened a space of one inch, which was occupied by an indurated mass extending as high as the internal ring, which are precisely the appearances of an ordinary inflammation of the testicle; this viscus forming the tumor, and the thickened vas deferens and spermatic cord the indurated mass between the tumor and the internal ring.

Vomiting is also a very frequent symptom of this inflammation, from the intimate sympathy between the stomach and testicle.

Under these circumstances, no mischief could ensue from waiting a few hours, till time had thrown more light on the nature of the case. If the bowels had been freely relieved by the enema administered in the interval, the existence of hernia would have been at once disproved; and, if the symptoms continued and hernia existed, the shortness of time could not make any material difference, as the want of severity in the symptoms would lead to the belief that the intestine had not sustained any serious injury. On visiting the patient at twelve P.M. and finding the symptoms, though not aggravated, yet remaining unabated, the operation was performed, to remove all doubt; and though, at the time of commencing it, I was not satisfied that the tumor was a hernia, yet I was conscious of doing right.

The remarkable appearances found in the operation were, the firm adhesion of the cremaster to the hernial sac, requiring considerable force to effect the passage of the director between them. On opening the sac, the omentum was in a state of gangrene, and would in a short time have sloughed off; it was therefore removed. The strictures, too, were peculiar: usually we find the stricture formed at the internal ring, but here were three distinct bands in the inguinal canal, each requiring to be divided. The adhesions between the sac, the intestine, and the omentum in the inguinal canal, are also worthy of notice; and, after separating these, the gangrenous spots were seen.

Some difference of opinion exists as to what course should be pursued in such cases: whether or not the whole should be left to the operations of nature. If the stricture be allowed to remain, the symptoms will continue till an opening in the intestine has been formed by gangrene; and, if we divide the stricture and intestine, we merely do that which nature would subsequently have effected, and by such a proceeding save invaluable time to the patient. This plan was therefore adopted.

After the operation, the Magnesiæ Sulph. in Aquæ Menth. Pip. was administered, as this is usually found to relieve the irritability of the stomach; and afterwards the colocynth enema was ordered, because the former enema had not been returned, and though there was a free discharge from the upper portion of the intestines, yet the distention of the colon might tend to keep up irritation. In the evening of the 7th, the bleeding was repeated, in order to guard against peritoneal inflammation, which occasionally takes place; and although the abdomen did not exhibit the symptoms of peritonitis, yet the pulse did.

At the time of the operation, it was impossible to ascertain what intestine was strangulated, whether jejunum or ileum; and in this doubt, as the powers of life sunk, it was conjectured as probable that the jejunum was involved, and that the aliment consequently escaped before a sufficient quantity had been absorbed. Beef-tea was therefore injected into the rectum, though less reliance is placed on this measure by practitioners in this country, than by the French and German surgeons. The subsequent treatment was merely the administration of increased nutriment.

The constitutional symptoms throughout this case were peculiar. We are told that, when gangrene exists, hiccough is a principal symptom; and that, in peritonitis, the abdomen is tense and extremely tender, and the pulse hard, sharp, and thready. All these symptoms were wanting in the present instance, and yet the intestine was gangrenous. The regular and progressive sinking was scarcely to be expected from the short interval which had elapsed since the strangulation. Much blood was not abstracted; and yet, at no one period after the symptoms of sinking presented themselves, did the patient's constitution exhibit the slightest disposition to rally. It is true that the vomiting latterly became less frequent, yet this appeared to arise merely from want of power.

From this case we should conclude that, when doubtful symptoms are present, an operation ought to be performed to ascertain the facts; and though in the present instance the termination was fatal, yet it would probably have been much earlier without an operation, and the surgeon would have been deservedly censured.

