# Errata

**Published:** 1827-01

**Authors:** 


					and la the present Number, page j3, line 20, read " extending nearly to."

				

